# Xeno-free production of lentiviral particles: implementing hPL as FBS replacement in cell culture

**DOI:** 10.3389/ftox.2026.1903400

**Published:** 2026-10-01

**Authors:** Mariana Soares Guedes, Marnie Cole, Daniela Yildiz

**Affiliations:** 1 Molecular Pharmacology, Center for Molecular Signaling (PZMS), Center for Gender-specific Biology and Medicine (CGBM), University of Saarland, Homburg, Germany; 2 Pharma Science Hub (PSH), University of Saarland, Homburg, Germany

**Keywords:** 3Rs (reduce, replace, refine), lentivirus, serum-free, virus production, serum-free adaptation

## Abstract

Commonly used for delivering fluorescent or bioluminescent reporters to target genes, lentiviral vectors are an “easy-to-clone” vehicle to study up- and downregulation of gene expression induced by drug inhibitors, cytokine stimulants, chemicals, and environmental toxicants. To produce viral particles safely, several plasmid constructs are delivered to human embryonic kidney cells (HEK293-T), which use the cellular machinery to package and assemble non-replicative lentiviruses with high efficiency. However, the cell culture of HEK293-T cells still largely relies on the use of fetal bovine serum for cell survival and proliferation. At large scale, this cell culture practice consumes a great amount of animal-derived serum, driving the need for continuous farming of animals whose fetuses would be used to obtain serum, posing grave ethical and practical concerns for human-relevant research. In this study, we present a feasible and scalable method to adapt the culture medium formulation for HEK293-T cells and demonstrate how virus production responds to this cell culture adaptation. Using a commercially available platelet-based supplement derived from human blood byproducts, we maintain cell viability and proliferation rates, with up to 82% confluence, and can produce lentiviruses (approximately 10^6^ infectious units/mL with up to 20% infectivity), while sparing the killing of bovine fetuses for serum collection, in a cost-efficient and humanized manner. Envisioning adoption in the pharma industry and large-scale virus production facilities, this simple yet robust cell medium formulation presents itself as an ethical and practical avenue to advance scientific innovation and bring one more layer of human relevance to biomedical research.

## 1 Introduction

Originally isolated from and studied in the context of animal diseases and human immunodeficiency, lentiviruses have been ingeniously re-engineered over the past several decades and revolutionized genetic engineering. Lentiviruses are enveloped, single-stranded RNA viruses belonging to the *Retroviridae* family, whose defining feature is the reverse transcription of their RNA genome into double-stranded DNA, which is subsequently integrated into the host cell chromosome (Durand and Cimarelli, 2011). This distinct capability allows lentiviruses to stably infect highly differentiated, post-mitotic cells, including neurons, macrophages, and hematopoietic stem cells, and has been thoroughly explored for biomedical applications (Gouvarchin Ghaleh et al., 2020).

Modern lentiviral vector (LV) systems are typically “pseudotyped” with the vesicular stomatitis virus G glycoprotein (VSV-G), which alters the natural tropism of the virus (Cronin et al., 2005). Native lentiviruses use the envelope glycoprotein gp120 to bind specifically to the CD4 receptor of immune cells, while VSV-G utilizes the low-density lipoprotein (LDL) receptor, which is highly conserved and expressed at the surface of almost all mammalian and non-mammalian nucleated cells (Finkelshtein et al., 2013). Thus, by replacing the native *Env* gene gp120/gp41 with VSV-G, LVs increase their viral tropism, as a “universal key” to infect both dividing and non-dividing cells across a vast spectrum (Farley et al., 2007). Additionally, the stable integration of cargo DNA into the host genome ensures long-term expression of transgenic material across successive cell generations. Standard applications encompass the delivery of fully functional genes to study gain-of-function, short hairpin RNAs for sustained gene knockdown and for CRISP/Cas9 delivery (Shalem et al., 2014; Adams et al., 2017; Li et al., 2018).

Beyond the laboratory setting, LVs have also been successfully integrated into clinical applications, such as *ex vivo* gene and chimeric antigen receptor (CAR) T-cell therapies, with nine prominent FDA-approved biologics currently on the market (Iglesias-Lopez et al., 2026). Rare genetic disorders can be targeted by obtaining a patient’s stem cells, using a lentiviral vector to insert a healthy copy of a defective gene, and infusing them back into the patient (Martínez-Molina et al., 2020). Because the gene will integrate directly into the patient’s DNA, the genetic correction is inherited by all downstream cell lineages, offering a lifelong cure. For CAR T-cell therapies, lentiviruses are used to reprogram a patient’s own T cells to express synthetic receptors targeted against tumor-specific antigens, helping the immune system to mount a robust cytotoxic response, achieving high rates of cancer remission (Labbé et al., 2021).

The human embryonic kidney 293 (HEK 293) cell line and its derivatives (such as HEK 293T) serve as the industry standard platform for producing recombinant LVs (Tan et al., 2021). To produce lentiviral particles safely, a four-plasmid transient transfection workflow is used to generate replication-deficient LVs (Dull et al., 1998; Robitaille et al., 2024). These plasmids carry i) *Gag-Pro-Pol*, which encodes viral structural proteins and enzymes necessary for virus packaging, ii) *Rev*, which expresses accessory proteins for viral genome export, iii) *Env*, which codes for envelope glycoproteins to allow cell receptor engagement and viral entry, and iv) the transfer vector coding for the gene of interest (Durand and Cimarelli, 2011; Merten et al., 2016). The HEK 293T cell line was developed by integrating the simian virus 40 (SV40) large T antigen into the genome of the parental HEK 293 cell line, allowing plasmids that carry the SV40 origin of replication to undergo replication when transfected into the cell (DuBridge et al., 1987; Tan et al., 2021). This modification increases the efficiency of transfection in this cell line and facilitates the generation of large titers of LVs.

As for most mammalian cell lines, culture and maintenance of HEK 293T cells rely on supplementing basal medium with a source rich in growth factors and hormones that stimulate cell proliferation, which can be found in both animal and human sera (Hutchings and Sato, 1978; Montali et al., 2016; Kamashev et al., 2023). Given its relative ease of production and high content of growth factors, fetal bovine serum (FBS) has therefore become the most extensively used supplement in cell culture (Meng and Day, 2026). Current estimates suggest that research, biotechnology, and pharmaceutical applications account for approximately 800,000 L of FBS per year, requiring the killing of approximately 2 million bovine fetuses worldwide (van der Valk et al., 2018; Pecora et al., 2020; Weber et al., 2025). This imposes clear limitations in the supply chain and creates bottlenecks and ethical concerns for the large-scale development of cellular therapies. Global regulatory frameworks are now strictly mandating a structural transition away from animal-derived components towards serum-free or chemically defined alternatives to guarantee the reproducibility, safety, and commercial viability of advanced gene and cell therapies (Ikebe and Suzuki, 2014; More et al., 2017; Weber et al., 2021; FDA, 2024).

Human platelet lysate (hPL), a byproduct of apheresis and buffy coats of healthy volunteers, has recently become a preferred human product alternative to FBS (Hemeda et al., 2014; Delabie et al., 2025). It is derived from human platelet units through the disruption of platelet membranes, releasing growth factors such as platelet-derived growth factors (PDGFs), basic fibroblast growth factor (b-FGF), vascular endothelial growth factor (VEGF), insulin-like growth factor 1 (IGF-1), and transforming growth factor beta (TGF-*β*) (Hwang et al., 1992; Doucet et al., 2005; Bernardo et al., 2007). hPL-cultured cells have been used for the treatment of graft-versus-host disease and for lumbar intervertebral degenerative disc disease, with no critical side effects reported (clinical trials: NCT01764100 and NCT01513694) (Introna et al., 2014; Gomez-Ruiz et al., 2023). The majority of studies evaluating hPL *in vitro* have initially focused on the expansion of human primary mesenchymal stromal cells (MSCs) for their use in human therapies in a GMP setting, such as bone marrow MSCs, adipose-derived MSCs, umbilical cord MSCs, and dental pulp MSCs (Burnouf et al., 2016; Guiotto et al., 2020). Additionally, primary and immune cells (T cells, NK cells, dendritic cells, dermal fibroblasts, HUVECs) and established tumor cell lines can be propagated in hPL (HEK293, HeLa, CHO, Vero, Jurkat, HepG2, HaCaT, A549, etc.), with studies reporting better proliferation rates than FBS and maintenance of functionality and signaling in different assays (Pons et al., 2019; Kolesnikova et al., 2026).

In light of these recent advancements, to allow an animal-product-free production of LVs, this study aimed to adapt HEK 293T cells to grow under hPL supplementation and to evaluate the efficiency of LV production under these conditions. To avoid effects on viability and virus production by direct replacement, we have selected a weaning strategy from FBS to hPL (Doucet et al., 2005; Bernardo et al., 2007). We were able to maintain comparable cell viability and proliferation rates, with up to 82% confluence, and produce lentiviruses (approximately 10^6^ infectious units/mL with up to 20% infectivity capacity). Thereby, the current study will stimulate an ethical and practical avenue to xeno-free scientific innovation and increase of human relevance in biomedical research practice.

## 2 Methods

### 2.1 Cell lines

Human embryonic kidney cells expressing SV40 large T antigen (HEK 293T) were cultured in Dulbecco’s Modified Eagle’s medium containing L-glutamine and sodium pyruvate (DMEM, Gibco, cat. # 41966029), supplemented with 10% heat-inactivated fetal bovine serum (cat. #10270-106, lot #2534396) and 1% penicillin/streptomycin (VWR, #3926459) in T75 cm^2^ flasks and kept in a humidified chamber at 37 °C with 5% CO_2_. At 80% confluence, cells were passaged at a 1/20 ratio by trypsinization using the animal-component-free trypsin TrypLE express (Gibco, #12604013). A defined trypsin inhibitor solution was used to terminate the TrypLE reaction (Gibco, #R007100).

### 2.2 Cell adaptation to human platelet lysate

To adapt the cell culture medium to FBS-free conditions, HEK 293T cells were initially seeded in 6-well plates at a density of 100,000 cells per well and allowed to attach for 4 h in medium containing 10% FBS. Once attached, cells were washed with 1x PBS, and the medium was changed to the compositions stated in Figure 1. The medium composition was maintained for 1 week, with regular passaging at 80% confluence, and a weekly adaptation over a period of 4 weeks. Two hPL formulations were tested as FBS replacement in this study: ELAREM™ Perform I (PL BioScience, cat. # PE10611, lot. nr. 21-017.01), requiring the addition of the anti-coagulant agent heparin (Braun, PZN 15782698, lot. nr. 2009212); and the fibrinogen-depleted, xeno-free human platelet lysate supplement ELAREM™ Perform-FD PLUS (PL BioScience, cat. #PE30621, lot. nr. 25-007.04) without the addition of heparin.

**FIGURE 1:**
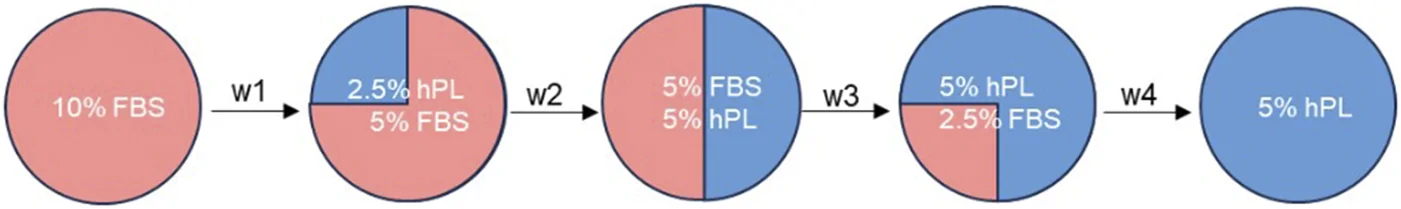
FBS to hPL medium adaptation schematic. The starting medium was composed of DMEM, 1% Pen/Strep, and 10% FBS. Cells were passaged two times a week upon reaching 80% confluence (usually at a 1:20 ratio), with medium changes performed every 2 days throughout the week. w, week; hPL, human platelet lysate; FBS, fetal bovine serum.

### 2.3 Live imaging kinetic assay

HEK 293T cells were seeded in 6-well plates as described in Section 2.2 and imaged in an automated microscope reader (Lionheart FX, Agilent) using a high-contrast brightfield filter and a ×4 objective. Cells were kept inside a humidified chamber stage insert at 37 °C and 5% CO_2_ throughout the kinetic imaging procedure. A total of five images were taken in different areas of each well to avoid seeding bias. The Gen5 Cellular Analysis feature of this microscope’s software permits the accurate identification of the cell outlines from high brightfield images, using the parameters set in Supplementary Table S1. Confluence was calculated from the sum of the masked area, divided by the total image area of 2.874,661 mm^2^ and multiplied by 100 to obtain a percentage, as per the yellow areas highlighted in Figure 2.

**FIGURE 2:**
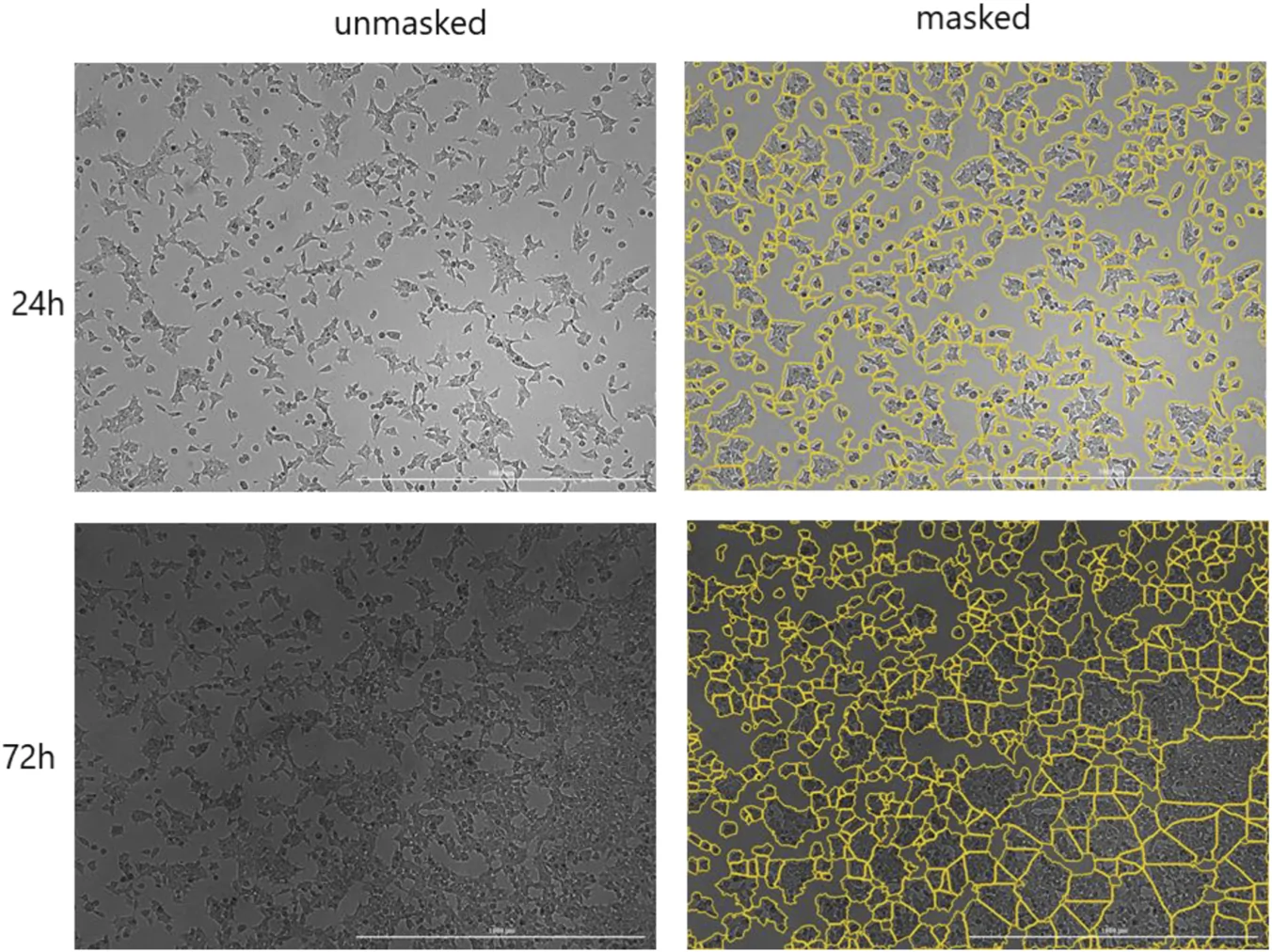
High-contrast brightfield imaging for confluence determination. Representative images of HEK 293T cells 24 h after seeding on 6-well plates and after 72 h of continuous imaging in the incubation chamber of the automated microscope. Original images on the left (unmasked) and with the confluence mask applied on the right panels (masked). Images acquired with a ×4 objective. Scale bar: 1,000 µm.

### 2.4 Lentivirus production

To produce lentiviral particles on adapted HEK 293T cells, the plasmids pMD2.G (3.75 µg, Addgene #12259), psPAX2 (8.75 µg, Addgene #12260), and pLVTHM scramble GFP (12.5 µg, from Aljohmani et al. (2022)) were co-transfected using JetPEI transfection reagent (Polyplus, # 101000020) following manufacturer’s standard protocol, in 6-well plates, with the cells at 60% confluence, in adapted cell medium containing 1% Pen/Strep. After 6 h, the medium containing the transfection mixture was replaced with fresh culture medium, and the cell supernatant was harvested 72 h later to collect the released viral particles by differential centrifugation. The collected supernatant was first centrifuged at 4,000 g for 10 min at 4 °C to remove cell debris, followed by syringe filtration using 0.45 µm PVDF filters and ultracentrifuged at 26,000 g for 2.5 h. The virus-containing pellet was finally resuspended in 100 µL of PBS with 0.02% sodium azide and stored at −80 °C in 20 µL aliquots until further use.

### 2.5 Quantification of transfection efficiency

Before harvesting viral particles from the cell supernatant at 72 h post transfection, each 6-well plate was imaged on a Lionheart FX microscope, using high-contrast brightfield for cell counting and the GFP channel for infected cell counting. Percent transfection efficiency was calculated by dividing the number of GFP-positive cells by the total cell count from each well.

### 2.6 Viral titer determination and nanoparticle analysis

Viral titers on stored virus aliquots were confirmed by qPCR using the Lentivirus titer kit following the recommended protocol by the supplier (abm, # LV900) and calculated using the abm online calculator sheet (*available at*: https://www.abmgood.com/qpcr-lentivirus-titration-titer-kit.html). In addition, physical characterization of the produced viral particles was conducted by nanoflow cytometry using the nano analyzer NanoFCM (NanoFCM Co., Ltd.). The instrument was adjusted before use with quality control silica beads to calibrate the optics and the filter and laser positioning. The stored viruses were thawed on ice, diluted 1:10 and 1:100 in sterile water, and passed through the NanoFCM capillary at a sampling pressure of 1.0 kPa for 1 min. Data processing to generate particle size and concentration was conducted directly on the NF Professional software (version 3.0). To discriminate between intact viral particles and viruses with damaged capsids, 1 µL of membrane-impermeable SYTOX Green nucleic acid dye (100 µM) was added to the 1:10 diluted virus samples and incubated in the dark at room temperature for 30 min before being passed through the capillary at the same sampling settings.

### 2.7 Determination of functional lentiviral titers

To assess the infectivity of the viruses generated under adapted conditions, 100,000 HEK 293T cells were seeded in a 12-well plate in medium containing 8 μg/mL of polybrene (Millipore, #TR-1003-G). Pooled virus aliquots from virus production experiments 1 and 2 were added to the medium for 24 h, after which the medium was refreshed without polybrene. Cells were imaged 48 h later using the automated microscope and quantified by counterstaining cell nuclei with DAPI (5 μg/mL) for total cell number count. The percentage of transduction was determined by the percentage of cells with GFP and DAPI co-localization versus the total number of DAPI-stained cells.

### 2.8 Statistical analysis

Unless otherwise stated, all experiments in this study were conducted twice, with a minimum of two technical replicates per experimental condition and experiment. Data are presented as mean ± SEM and were analyzed in GraphPad Prism 8.0 software. Significance in Figure 3C was determined by one-way ANOVA with multiple comparisons. The difference was considered statistically significant for p-value <0.05 and represented as follows: *, *p* < 0.05; **, *p* < 0.01; ***, *p* < 0.001; and ns, non-significant.

**FIGURE 3:**
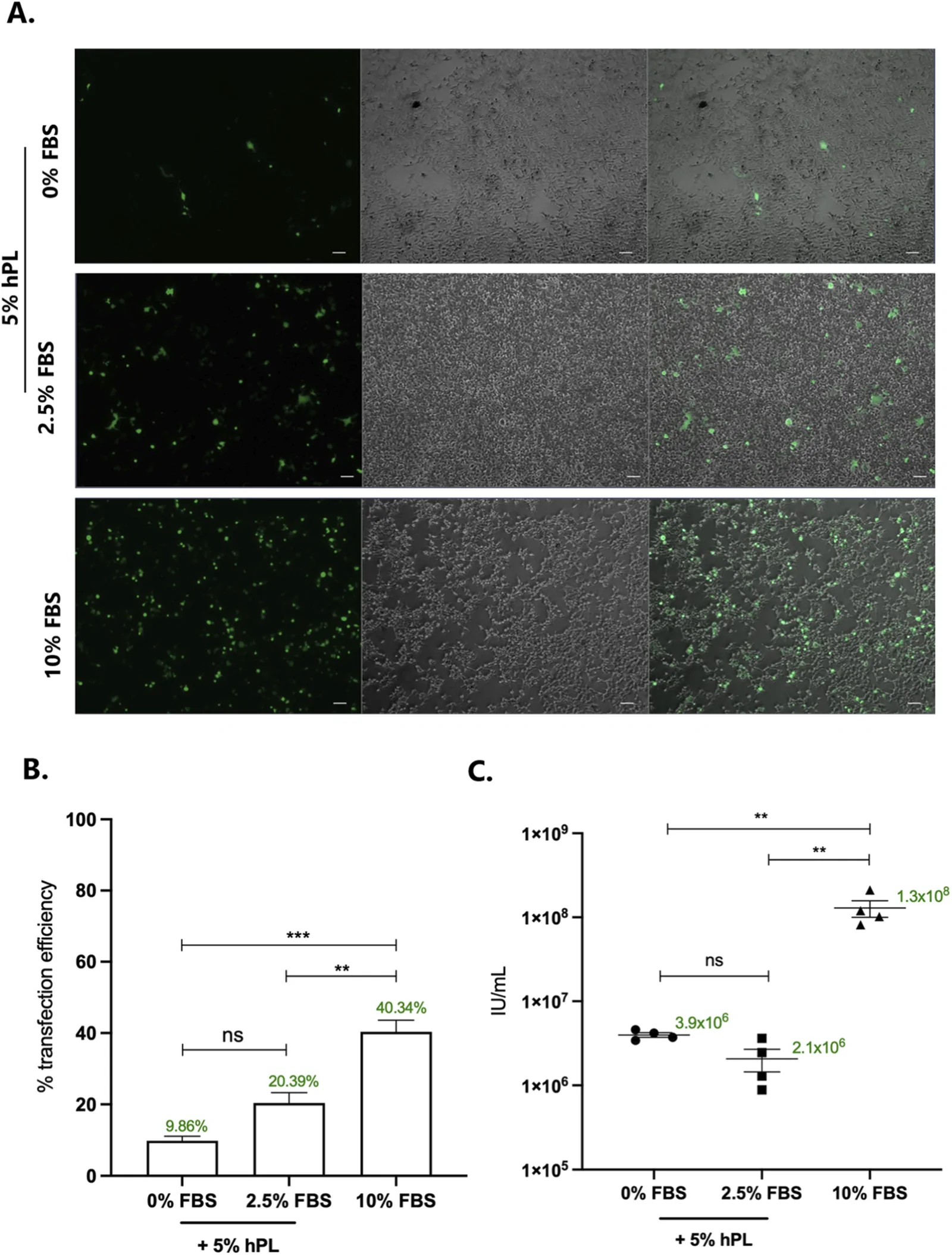
Lentivirus production and titers in HEK 293T grown with hPL or FBS. **(A)** Representative GFP fluorescent and phase contrast images of the transfected HEK 293T cells at 72 h post transfection. ×32 objective; scale bar: 100 µm. **(B)** Transfection efficiency was determined on a Lionheart FX automated microscope by analyzing the entire area of the wells in all conditions. **(C)** After purification and concentration by ultracentrifugation, viral titer was determined using a lentiviral qPCR kit to quantify the infectious units (IU) per milliliter. In **(B,C)**, the green values represent the average % transfection efficiency and IU/mL, respectively.

## 3 Results

### 3.1 Human platelet lysate supplementation supports the growth and proliferation of HEK 293T cells

To gently adapt any immortalized mammalian cell line to FBS-free culture conditions, a slow approach is preferred by decreasing the amount of fetal bovine serum over time, while increasing the serum-free replacement supplement. Using this approach, we were able to generate a HEK 293T cell line fully adapted to FBS-free growth and maintenance in a 1-month time span. We first started by reducing the amount of FBS to hPL at 100/0, 75/25, 50/50, 25/75, and finally, 0/100 ratios. Interestingly, we rapidly noticed that the cells on the 50/50 adaptation step grew two to three times faster than in 10% FBS cells, shrank significantly in size, and consequently detached very easily as a single monolayer within less than 36 h of culture, affecting the viability of cells to proceed to the next 25/75 adaptation ratio (*data not shown*). Therefore, we chose a final concentration of 5% hPL, as the growth dynamics or cell morphology at higher concentrations were not comparable to 10% FBS supplementation. We then proceeded with the strategy shown in Figure 1. Cells were kept in culture and continuously passaged for an additional 3 months, cryopreserved in 5% hPL-containing cell culture medium with 10% DMSO, and successfully recovered before performing any further analysis. An important technical note is the specification of the hPL subtype. The ELAREM™ Perform I formulation, requiring the addition of 1 unit (U/mL) of heparin in the cell medium composition, prevented the cells from attaching to the cell culture plates, even when the wells were pre-coated with collagen I or poly-L-lysine (*data not shown*). The ELAREM™ Perform-FD PLUS, however, does not require the addition of heparin to the cell culture medium and resulted in adherent monolayers (Figure 3A).

To quantify cell growth over time, we performed kinetic live imaging on an automated microscope over the course of 3 days and determined the cell confluence for HEK 293T grown with 10% FBS, 5% hPL+2.5% FBS, or 5% hPL supplementation (Figure 3A and Supplementary Videos S1-S3), seeding the cells at equal cell numbers. Cells grown with 10% FBS or 5% hPL+2.5% FBS followed a prototypical sigmoidal proliferation curve, with an initial lag phase of approximately 12 h, followed by steady proliferation until 103.5 ± 21 h for 5% hPL+2.5% FBS (average peak confluence: 63.25% ± 19%) and nearly 130 h for 10% FBS (average peak confluence of 82.6 ± 7.2%) (Figure 3B). On the other hand, cells grown under 5% hPL presented a less pronounced lag phase and continuous steady proliferation peaking at 112.5 ± 17 h, reaching a comparable confluence to 10% FBS (average peak confluence: 81.6 ± 8.4%). However, further growth resulted in a decline in cell confluence, not due to cell death but observable by cell shrinkage (Figure 3C). To allow a rapid transfer of the xeno-free culture to other laboratories, we tested a direct switch to FBS-free culture instead of a weaning strategy. However, we observed a strong effect on cell growth and viability data not shown, which led to the decision to proceed with the weaning strategy.

### 3.2 Lentivirus production in HEK 293T cells is impacted by hPL supplementation

HEK 293T cells are commonly used for pharmaceutical and R&D applications for LV production due to their fast proliferation and permissive and robust transfection. Given the similar growth patterns and dynamics of the three supplementation strategies, we next assessed the capacity of the adapted cells to produce viable LV particles for downstream applications. We found striking differences in both transfection efficiency and number of infectious units (IU) per mL between hPL supplemented versus FBS supplemented cells, with maximum efficiency at 20.4 ± 2.82% for 5% hPL+2.5% FBS supplementation (combined average of 3 x 10^6^ ± 9.6 × 10^5^ IU/mL between 5%h PL and 5% hPL+2.5% FBS) and 40.3 ± 3.2% with average 10^8^ IU/mL on 10% FBS supplementation (Figure 4).

**FIGURE 4:**
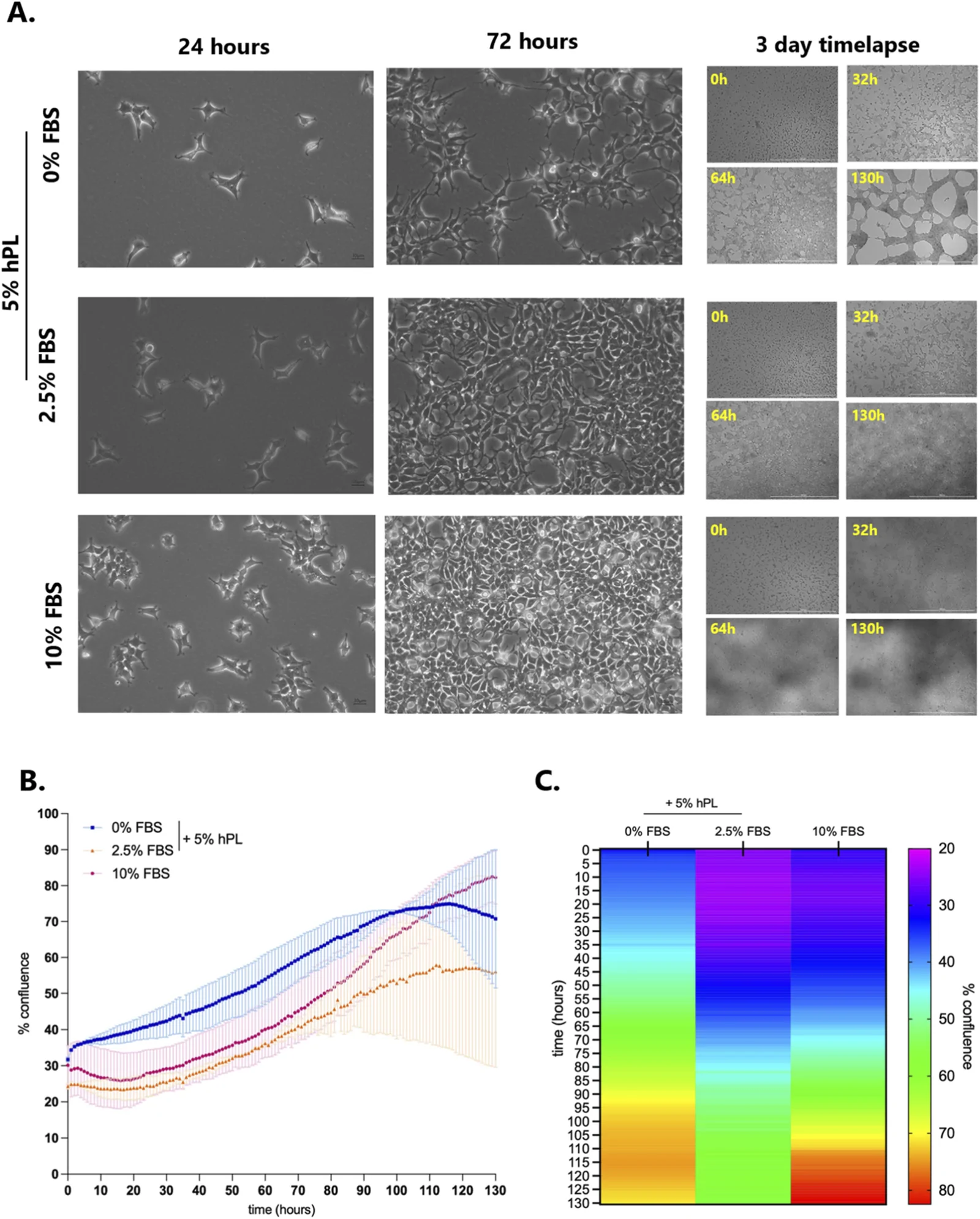
HEK 293T cell growth dynamics with human platelet lysate supplementation as FBS replacement. **(A)** Representative phase contrast images under a ×20 objective were acquired at 24 h and 72 h post-seeding HEK 293T cells. Scale bar on phase contrast: 20 μm **(B,C)**. Representative high-contrast brightfield images, captured at ×4 magnification (brightfield timelapse: 1,000 µm) from one of five locations on the well plate, were used to calculate the percentage of confluence over 130 h using the Gen5 software (N = 2 experiments, two replicates per condition). Please refer to Supplementary Videos S1–S3 for increased resolution. Data in **(B)** represent the mean values ± SEM. In **(C)**, a heatmap was generated for better visualization.

### 3.3 Downstream characterization of virions and infectivity capacity reveals a potential for large-scale LV production with hPL-supplemented HEK 293T cells

Nanoflow cytometry (NanoFCM) is a rapid solution for obtaining information about LV titer, purity, and empty/full ratio, facilitating different stages of large-scale virus production, purification, quality control, and stability assessment. At first, we analyzed the cell medium composition at 80% confluence of the three supplementation regimens by NanoFCM (Supplementary Figure S1). While the particle concentration was similar in all conditions, we detected clear differences in the size of the particles in the medium of the cells grown with 5% hPL (average size ± 150 nm) versus cells grown solely with 10% FBS (average ± 100 nm). Then, to evaluate the reproducibility of our xeno-free approach, we analyzed the viruses collected in two independent experiments of virus production by NanoFCM and characterized them under label-free and nucleic acid-stained conditions by using a non-membrane-permeable fluorescent dye. LVs were found within a range of 80–120 nm, as expected, with a larger number of contaminating particles on viruses purified from hPL-supplemented cells (Figure 5A). Nevertheless, the percentage of intact virions was similar in all cases (average of 72.71 ± 8.6%, Figure 5B), indicating that, despite being produced at lower levels, the virions remained unaffected by hPL components during the cell-mediated virus assembly stage.

**FIGURE 5:**
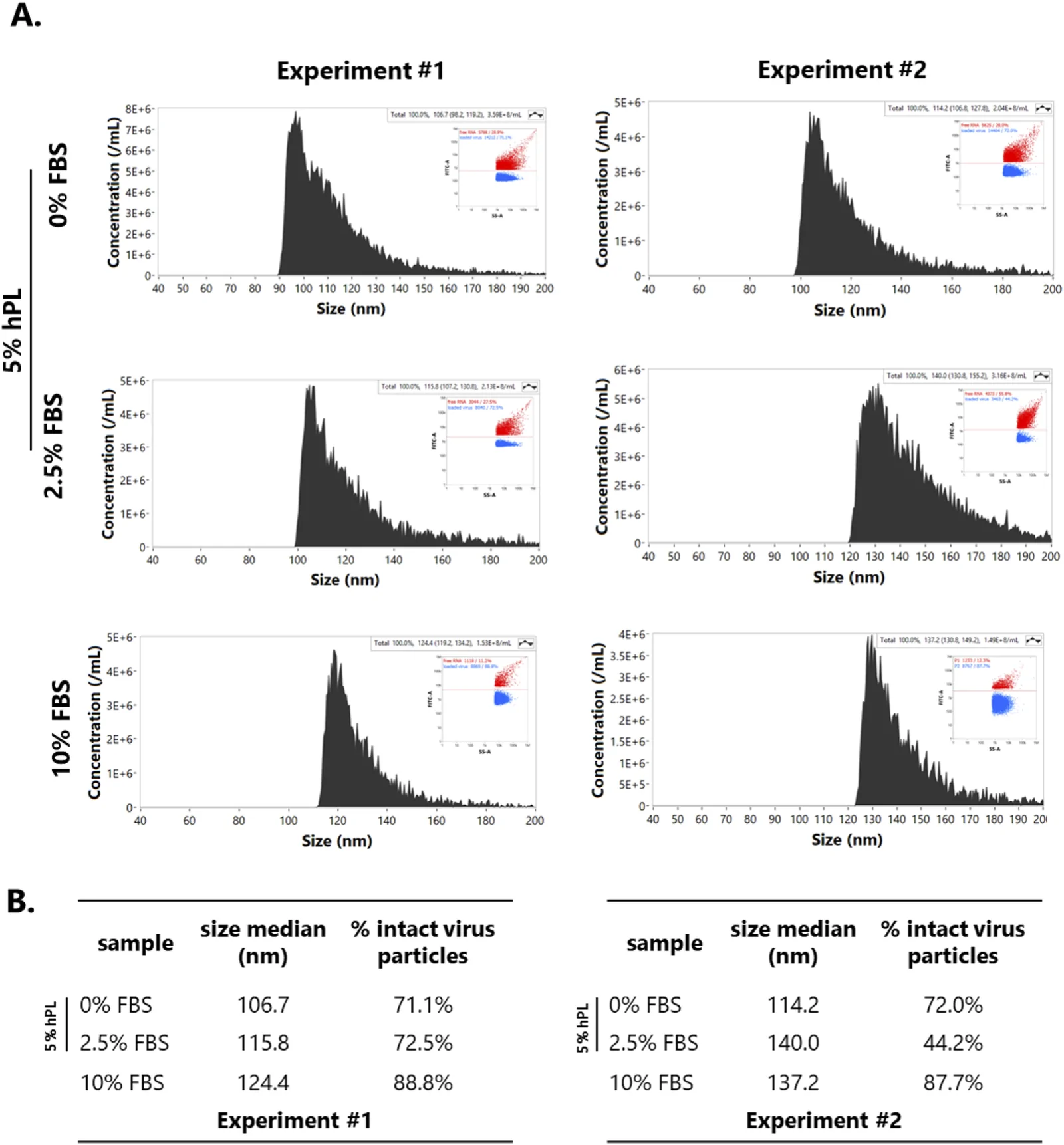
Nanoparticle content analysis of LV production under 5% hPL supplementation. **(A)** Nanoflow cytometry plots of nanoparticle size distribution and concentration for two independent virus productions, with two replicates each (experiment 1 and experiment 2). Colored scatter plots indicate the percentage of non-viable viruses (red: SYTOX Green stained) versus intact viruses (blue: unstained). See Supplementary Figure S2 for higher-resolution cutouts. **(B)** Quantification of the data presented in **(A)**.

Based on the IU/mL measurements from our previous analysis and the relatively small scale of our virus production experimental setup, we pooled the viruses produced in experiments 1 and 2 and infected HEK 293T cells at a multiplicity of infection (MOI) of 1 (Figure 6A). We observed a clear reduction in the GFP signal after 48 h in the LVs produced from cells grown with 5% hPL (mean 8.81% ± 2.2% and 14.55 ± 1.35% for no FBS and 2.5% FBS, respectively) compared with an average of 59.35 ± 1.69% for the 10% FBS counterpart (Figure 6B).

**FIGURE 6:**
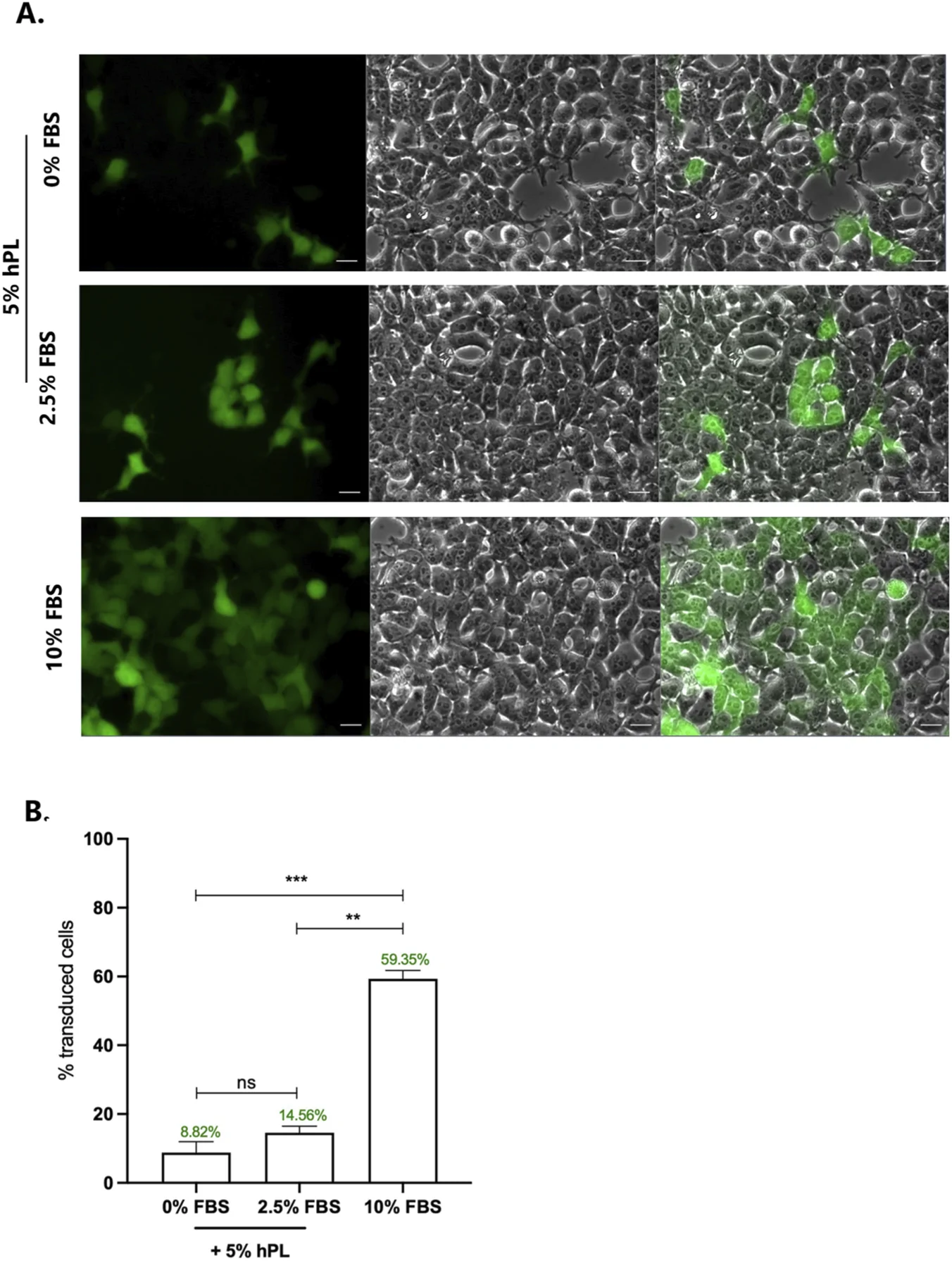
Transduction efficiency and infectivity of LVs produced with hPL-adapted HEK 293T cells. **(A)** Representative GFP fluorescent and phase-contrast images of the transfected HEK 293T cells at 48 h post transduction. ×32 objective; scale bar: 100 µm. **(B)** Quantification of the percentage of transduced cells (average % values highlighted in green) assessed per whole-well imaging of DAPI-stained nuclei and fluorescent green cells. This experiment was done once, with two replicates per condition.

## 4 Discussion

In this paired culture study, we showed that 5% hPL is a suitable xeno-free supplement alternative for 10% FBS in HEK 293T proliferation and maintenance. We detected no significant alterations in the morphology of the cells and identified conditions in which the cells sustained similar growth rates as for the established 10% FBS supplementation. However, the ability to produce lentiviruses was reduced by two log-fold in cells supplemented with hPL, underscoring the importance of evaluating the cell-type-specific effects of hPL in different cell models and respective downstream applications.

Previous studies have shown that, despite the similarity in the overall protein, hormone, growth factors, and ion contents between FBS and hPL, the concentration ranges of these components vary greatly, with IGF and cortisol being two-fold higher in hPL (125 μg/L, 72.8–77.3 μg/L) than FBS (40–72 μg/L, 20 μg/L) (Burnouf et al., 2011; Burnouf et al., 2012; Shanskii et al., 2013; Meng and Day, 2026). Additionally, hPL has a higher content of immunoglobulins, cytokines, protease inhibitors, and the possible presence of coagulation factors when produced without thrombin activation, which influence cell growth, differentiation, and function (Horn et al., 2010).

Recently, key differences in integrin signaling were observed in the non-small cell lung carcinoma HCC827 line when grown under hPL supplementation, which were absent when other lung cell lines were grown in the same conditions (i.e., A549 or H1299) (Woitaske-Proske et al., 2026). Likewise, we observed that HEK 293T cells followed similar morphological changes under FBS-free conditions (5% hPL only), namely, significant reorganization, forming gaps between cell clusters and exhibiting a stretched phenotype, consistent with cytoskeletal architecture changes, as observed in previous studies (Faussner et al., 2022; Delabie et al., 2026; Kolesnikova et al., 2026). This effect was completely abolished when a very small amount of FBS (2.5%) was present in the culture medium. Nevertheless, when combining hPL with FBS (in this case, 5% hPL with 2.5% FBS), we observed a delay in cell growth and a lower maximum confluence of 60%. Although counterintuitive, the addition of a bovine supplement alongside the human-derived proteins might induce xenogeneic cell stress in combination with increased amounts of TGF-β, which, on its own, can act as a cell cycle inhibitor for epithelial cells at high concentrations (Hannon and Beach, 1994; Zhang et al., 2017). Chromatographic analysis could be a valuable tool to pinpoint the exact components present in both conditions and determine which proteins or metabolites are inducing these morphological changes. Such investigation would allow for a factor-specific supplementation of hPL to minimize cellular effects and to increase the suitability for downstream applications such as LV production (Palombella et al., 2022; Delabie et al., 2025; Immalaraju et al., 2025).

With regard to the impact of serum on the yield performance of lentiviruses, including transfection efficiency and viral particle functionality, there is a lack of comparable studies using hPL in the literature. A reduction of 50% in LV titers was observed when transitioning from adherent HEK 293T culture in serum-containing medium to a serum-free suspension culture model, which required intensive optimization of transfection protocols and alternative lentiviral vector payloads to improve the number of viral infective units (Bauler et al., 2020). Likewise, engineered HEK 293T cells producing a secreted nuclease to reduce the need to add exogenous nuclease to purified virus preparations failed under serum-free conditions in morphology, growth dynamics, and viral titers compared to the parental cell line grown in FBS-containing medium (Ali et al., 2023). Our analysis of the purified viruses by nanoflow cytometry revealed a much higher microvesicle and exosome content in viruses obtained from hPL-packaging cells than in FBS-supplemented cells. Given that the transfection agent used in this study is based on micelle formation (Gallops et al., 2020; Jogdeo et al., 2024), the detected higher content of nanoparticles in hPL could interfere with membrane interaction and vector DNA uptake capacity of these cells. Furthermore, fusion of these nanoparticles with micelles could be an additional explanation for the low transfection efficiency. Therefore, ultracentrifuged and nanoparticle-depleted hPL could be a solution to be considered in future studies.

Lentiviruses are enveloped particles with a diameter of roughly 80–120 nm, while extracellular vesicles (EVs) and exosomes fall into a nearly identical size bracket of 30–150 nm, sharing very similar surface charges and buoyant densities (Flaujac et al., 2009; McNamara and Dittmer, 2020; Do Minh et al., 2021). FBS naturally teems with an immense concentration of EVs, and it is one of the main reasons why it is avoided for clinical applications, with a high rate of reported immunological reactions against xenogeneic serum antigens (Sakamoto et al., 2007; Sundin et al., 2007). On the other hand, platelet-derived exosomes dynamically impact LV infectivity given their capacity for endocytosis or encapsulating LVs within their lipid bilayers, which effectively conceals viral envelope proteins from their target host cell receptors (Soriano-Sarabia et al., 2022; Kushch and Ivanov, 2023). Moreover, when lentiviral particles are internalized and come into contact with specialized platelet-derived EV components or alpha-granule content, the virus can undergo morphological degradation and lose its outer envelope, permanently destroying its infectivity, thus potentially increasing the number of defective particles in LV preparations (Flaujac et al., 2009).

In addition to soluble factors and nanoparticles, differences in the lipid composition of FBS and hPL could further change the biophysical properties and raft formation of the HEK293 T cells. Thus, future investigations on FBS alternatives should not only focus on the proteome and metabolome differences, but also on lipid composition and biophysical aspects (Ali et al., 2026; Koelz et al., 2026; Zhang et al., 2026). Bramwell et al. (2024) presented a comprehensive stepwise protocol guidance for transitioning five different human primary cell lines to animal-component-free (ACF) culture, resorting to human serum. However, the use of human serum not only poses a supply chain bottleneck but remains a highly variable and poorly characterized supplementation strategy that does not work for all cell lines. To this end, the adoption of a chemically defined medium composition presents a more reliable and reproducible solution, given that all components of the medium can be titrated and manipulated individually to evaluate their impact on specific cellular processes. The Oredsson Universal Replacement (OUR) medium is one such solution that has been made publicly available with detailed guidance and protocols for preparing this medium formulation, to expand the further adoption of ACF-defined medium to an increasing number of human cell lines (Rafnsdóttir et al., 2023; Weber et al., 2024; Malakpour-Permlid et al., 2025; Oredsson et al., 2025). Additionally, the fetal calf serum-free database is a curated open-source search tool for commercially available alternatives to FBS and animal-free medium compositions described in the literature, which provides a great starting point for any research looking to transition to humanized cell culture practices (Utrecht University, 2017). For HEK293 T cells, for example, only one publication has been reported for a chemically defined medium, one serum-free adapted HEK293 T-cell line, and one commercial supplement is currently available and tested for this specific cell line, thus bypassing some of the laborious guesswork and empirical evaluation that would otherwise have been necessary (Kuroda et al., 2009; Kulkarni et al., 2024; Nanami Kadota et al., 2024). Nevertheless, implementation in academic settings of fibrinogen-depleted hPL remains 2× to 5× more costly per liter than FBS. Because it necessitates mandatory donor screening, pathogen reduction/inactivation, and pooling, it significantly adds to the regulatory overhead that inflates end-user pricing. In practical terms, the lower-cost fibrin-containing hPL formulations require the use of heparin, which is still porcine derived, introducing a non-human product to the xeno-free system (Kee et al., 2023).

At present, there is a clear lack of consensus and standardization when it comes to FBS-free alternative culture models, which hampers a broader adoption of animal-free research methodologies (Immalaraju et al., 2025). Nevertheless, we hope that this work provides a solid basis for future investigations, in which commercial and academic groups, such as our own, can apply this approach to animal-component-free large-scale virus production and purification. Ideally, this work will stimulate joint efforts of the community to develop chemically defined media, suitable for LV production at large scale.

## Data Availability

The original contributions presented in the study are included in the article/Supplementary Material; further inquiries can be directed to the corresponding author.
