# Supplementary material for: Xeno-free production of lentiviral particles: implementing hPL as FBS replacement in cell culture
Source: Front Toxicol. 2026 Oct 1;8:1903400. doi: 10.3389/ftox.2026.1903400 (PMC13669877; doi:10.3389/ftox.2026.1903400)
Supplement: Supplementary file 1 [file DataSheet1.docx]

Supplementary Material

**A xeno-free approach to lentivirus production – staving off FBS in standard cell culture practices**

Mariana Soares Guedes^1^<https://orcid.org/0000-0002-5549-0620>, Marnie Cole^1^, Daniela Yildiz^1,2*^<https://orcid.org/0000-0002-9702-8581>

^1^ Molecular Pharmacology, Center for Molecular Signaling (PZMS), Center for Gender-specific Biology and Medicine (CGBM), University of Saarland, Germany.

^2^ Pharma Science Hub (PSH), University of Saarland, Germany.

*** Correspondence:**
Daniela Yildiz
daniela.yildiz@uni-saarland.de

**Contents:**

**Figure S1** – Nanoparticle content analysis of cell culture medium with and without hPL.

**Figure S2** – Scatter plots of intact vs. damaged viral particles (related to Figure 5).

**Table S1** – Cellular analysis parameters used on Gen 5 software to generate masked images to track confluency over time. *Related to Figure 1.*

**Video S1** – Timelapse video recording of HEK 293T grown with 5% hPL supplement and no added FBS. Images were acquired every 30 minutes for a total of 130 hours.

**Video S2** – Timelapse video recording of HEK 293T grown with 5% hPL supplement and 2.5% FBS. Images were acquired every 30 minutes for a total of 130 hours.

**Video S3** – Timelapse video recording of HEK 293T grown with 10% FBS supplementation. Images were acquired every 30 minutes for a total of 130 hours.

**Figure S1 –** **Nanoparticle content analysis of cell culture medium with and without hPL.** Size and concentration distribution of small particle content obtained with nanoflow cytometry on 20 µL of cell culture medium under 5% hPL, with or without 2.5% FBS and with 10% FBS only.

**Figure S2** – **Scatter plots of intact vs. damaged viral particles analyzed by NanoFCM** (related to Figure 5). Plots represent the stained viruses with damaged capsids (FITC-A) in relation to the total particle population (side scatter-Area).

Table S1 – Cellular analysis parameters used on Gen 5 software to generate masked images to track confluency over time. *Related to Figure 1*.

| **Cellular Analysis Parameters** | |
| --- | --- |
| Channel | Bright Field |
| Threshold | 1000 |
| Background | Light |
| Split touching objects | Checked |
| Fill holes in mask | Checked |
| Min. object size | 30 µm |
| Max. object size | 3000 µm |
| Include primary edge objects | Checked |
| Analyze entire image | Checked |
| **Advanced Analysis Parameters** | |
| Rolling Ball diameter | 20 µm |
| Image smoothing strength | 5 cycles of 3x3 average filter |
| Evaluate background on | 0% |
| Primary mask | Use threshold mask |
